# Effect of expanding farmlands with domestication of animals in the vicinity of disturbed swamps and built-up farmland ponds on population dispersion and decline of locally adapted *Mansonia* vectors (*Diptera*: *Culicidae*)

**DOI:** 10.14202/vetworld.2024.564-576

**Published:** 2024-03-08

**Authors:** Suntorn Pimnon, Adisak Bhumiratana, Apiradee Intarapuk, Wanapa Ritthison

**Affiliations:** 1 Faculty of Public Health, Bangkokthonburi University, Bangkok 10170, Thailand; 2 Thammasat University Research Unit in One Health and EcoHealth, Pathum Thani, Thailand; 3 Faculty of Public Health, Thammasat University, Rangsit Campus, Pathum Thani 12121, Thailand; 4 Faculty of Veterinary Medicine, Mahanakorn University of Technology, Bangkok 10530, Thailand; 5 Office of Disease Prevention and Control 6 Chonburi, Thailand

**Keywords:** built-up farmland ponds, disturbed swamps, expanding farmland, freshwater habitats, and animal blood meal sources, *Mansonia* fauna

## Abstract

**Background and Objectives:**

The adaptive processes and resilience of *Mansonia* vectors responsible for bioindicators can change in response to climate, land use, and environmental changes. This study evaluated the effects of expanding farmlands with the domestication of animals in the vicinity of either disturbed swamps or built-up farmland ponds on the population dispersion and decline of locally adapted *Mansonia* faunas as a result of expanding farmlands in Thailand.

**Materials and Methods:**

Based on environmental surveys, four different geographically defined study sites were selected: I - the expanding farmlands with domestication of livestock and pet animals in the vicinity of low-lying swamp with habitat fragmentation and aquatic vegetation; II - the expanding farmlands with domestication of pet animals in the vicinity of elevated swamp with habitat destruction and aquatic vegetation; III - the expanding farmlands with domestication of livestock and pet animals in the vicinity of low-lying farmland ponds with restoration and aquatic vegetation; and IV - the expanding farmlands with domestication of pet animals in the vicinity of elevated farmland ponds with restoration and aquatic vegetation. Human landing catch collection method was used to periodically assess the species composition and abundance of *Mansonia* vectors.

**Results:**

Aggregated distributions and seasonal abundances of *Mansonia* faunas (*Mansonia uniformis*, *Mansonia indiana*, *Mansonia annulifera*, *Mansonia annulata*, *Mansonia bonneae*, and *Mansonia dives*) with variable proportions were observed at all the study sites. A decline in the population of *Mansonia* faunas, except for *Ma. uniformis*, was observed at study sites I and II.

**Conclusion:**

The anticipated effects of expanding farmlands affected the population dispersion and decline of locally adapted *Mansonia* faunas, thus representing a diverse assemblage of *Mansonia* species with different adaptations, ecological tolerances, and host exploitation strategies in life. These effects depended either on the function of disturbed swamps or on the development of farmland ponds, whether they provided a wide range of freshwater habitats, or on the domestication of animals, whether they provided animal blood meal sources.

## Introduction

*Mansonia* mosquitoes (*Diptera*: *Culicidae*), belonging to the genus *Mansonia*, are known to be vectors for arboviruses [1–3] and lymphatic filarial nematode parasites *Brugia malayi* [4–7] and *Wuchereria bancrofti* [8, 9]. *Mansonia* is a strong zoophilic species that can attack livestock, poultry, and wild or domestic animals. Certain *Mansonia* spp. are major nuisance pests. Due to their seasonal abundance, they become more pestiferous to livestock and humans [2, 4, 10]. *Mansonia* fauna (i.e., all *Mansonia* species present in a particular area or time) is sessile to a wetland ecosystem at a particular altitude over time. *Mansonia* faunas that infest freshwater/blackish habitats across the globe are classified into two subgenera: *Mansonia* Blanchard and *Mansonoides* Theobald [11, 12]. Understanding the population dispersion, abundance, and spatial/temporal distribution of *Mansonia* fauna is crucial in establishing the importance of veterinary public health regarding nuisance pests to animals and the potential in the transmission dynamics of pathogens to animals and humans. The subgenus *Mansonia* consists of approximately 15 species [11, 12]: *Mansonia amazonensis*, *Mansonia cerqueirai*, *Mansonia chagasi*, *Mansonia dyari*, *Mansonia flaveola*, *Mansonia fonsecai*, *Mansonia humeralis*, *Mansonia igaussuensis*, *Mansonia indubitans*, *Mansonia leberi*, *Mansonia pessoais*, *Mansonia pseudotitillans*, *Mansonia suaresi*, *Mansonia titillans*, and *Mansonia wilsoni*; they are most commonly found in marshy areas in tropical countries in the Americas [2, 3, 13, 14]. *Ma. titillans* (Walker), an important pest in Central and South America [2, 3, 15], is known to transmit various arboviruses, including Venezuelan equine encephalitis [2], while *Ma. dyari* (Belkin, Heinemann, and Page) is considered a potential vector of Rift Valley fever virus [1]. The subgenus *Mansonoides* contains approximately 10 species [11, 12]: *Mansonia africana*, *Mansonia annulata*, *Mansonia annulifera*, *Mansonia bonneae*, *Mansonia dives*, *Mansonia indiana*, *Mansonia melanesiensis*, *Mansonia papuensis*, *Mansonia septempunctata*, and *Mansonia uniformis*. Among these, *Ma. uniformis* (Theobald), *Ma. annulata* (Leicester), *Ma. annulifera* (Theobald), *Ma. indiana* (Edwards), *Ma. bonneae* (Edwards), and *Ma. dives* (Schiner) are known to be natural vectors for Malayan filariasis caused by the nocturnal sub-periodic *B. malayi* in endemic countries in Southeast Asia, including Indonesia [5, 16, 17], Malaysia [6, 7, 18–20], and Thailand [21–24]. Previous studies demonstrated that certain *Mansonia* exophilic species exhibit endophilic hematophagy, particularly when expanding human populations invade their natural habitats, particularly in *B. malayi* endemic villages in the vicinity of swamps [4, 7, 9, 10, 16–19, 24]. Among these *Mansonoides* mosquitoes, *Ma. uniformis*, *Ma. annulifera*, and *Ma. indiana* are considered indicator species that can disperse through tropical countries in SEA, South Asia, such as India [4, 25, 26], Sri Lanka [27], and South China [28].

Most studied faunas of *Mansonia* (*Mansonoides*) mosquitoes are native to swamps and marshes. If vegetated with floating-leaf or emergent aquatic macrophytes, these freshwater habitats allow *Mansonia* to breed larvae in stagnant and turbid water with an earthy mixture [23–27, 26, 29, 30, 31]. The preferred aquatic macrophytes that serve as ovipositional sites include water hyacinth, *Monochoria hastata* (L.), Solms var. hastata or *Eichhornia crassipes* (Mart.) Solms, water lettuce *(Pistia stratiotes*), and salvinia, either *Salvinia molesta* or *Salvinia cucullata* Roxb, but not cattails, water mimosa, hydrilla, duckweed, or dwarf waterclover. These aquatic host plants can provide mechanical support to the larvae by attaching their roots to obtain air supply and protect them from physical disturbances, predators, and sun exposure. The world is expected to face severe droughts, whether meteorologically, hydrologically, agriculturally, or socioeconomically, likely due to El Nino [32, 33]. It is the most serious hazard to livestock, crops, and people’s livelihood. This uncertainty effect of El Nino is associated with an unprecedented phenomenon, i.e., whether flooding or drought is expected to have health and well-being effects through reduced water quantity, food security, and drought-related vectors in the present or future [32, 33, 34, 35]. Thailand is vulnerable to intense drought [36, 37], leading to a decrease in precipitation during the rainy season. Therefore, an efficient farmland irrigation system and adequate water storage are essential to mitigate droughts [38]. As farmers and landowners expand farmlands, the restoration of farmland ponds and ditches is vital to help prevent future drought problems on farmland and alleviate ecological service functions that support the hydrological cycle for agriculture and domestic uses [37, 38]. However, farmers and landowners did not pay much attention to the impact of the expansion of farmland with the domestication of animals, whether establishing or re-establishing habitats of *Mansonia* vectors, while maintaining the restoration of farmland ponds.

The objective of this study was to evaluate the effects of expanding farmlands with domestication of animals in the vicinity of swamps with habitat fragmentation/destruction or farmland ponds with restoration and aquatic vegetation on the population dispersion and decline of locally adapted *Mansonia* faunas by periodically assessing the species compositions and abundances of *Mansonia* faunas in four different geographically defined study sites in southern and eastern Thailand. This would help to understand how the population of *Mansonia* faunas locally adapted to freshwater ecosystems over time due to the anticipated effects of expanding farmlands and help to target surveillance and control activities in farmland areas where *Mansonia* faunas are responsible for natural vectors of filarial parasites and arboviruses are invading or establishing/reestablishing habitats.

## Materials and Methods

### Ethical approval

The Institutional Review Board of the Faculty of Veterinary Medicine, Mahanakorn University of Technology (Approval No. ACUC-MUT-2014/001), and the Faculty of Public Health, Mahidol University (COA No. MUPH 2016-008) approved the ethical clearance for this study.

### Study period and location

The study was conducted from March 2014 to May 2017 in Narathiwat, Suratthani, and Trat Provinces.

### Environmental surveys and study site selection

Multidisciplinary field teams performed environmental surveys to map the spatial distribution of farmlands, freshwater habitats of *Mansonia*, and domestication of animals using global positioning system tracking of locations and Google Earth Pro version 7.1 application for managing geographical information system data (https://www.google.com/earth/about/versions/#download-pro). Since the destruction of wetlands in Thailand has so far been recognized, survey areas covering expanding farmlands were initially selected based primarily on the report of an inventory of wetlands in Thailand by the Office of Environmental Policy and Planning, Ministry of Science, Technology, and Environment, Thailand [38] and the previous studies of *Mansonia* fauna in freshwater habitats since 2002 [24, 29, 39]. The Universal Transverse Mercator (UTM) coordinates of the survey farmland area were measured as eastings (m E) and northings (m N). Therefore, it was possible to select each survey farmland area demarcated within a surface area of 4 km^2^ as a study site. Subsequently, the multidisciplinary field teams carefully examined each study site on foot, identifying and mapping potential freshwater habitats of *Mansonia*, and completing thorough veterinary public health inventories, including farmlands with domestication of animals (whether livestock or pet animals) in the vicinity of disturbed swamps or built-up farmland ponds with the restoration and vegetation of aquatic host plants. These surveys were conducted at time intervals during the year to adequately observe the abundance distributions of *Mansonia* female adult mosquitoes and larvae before the study. The four study sites are described below.

**Figure-1:**
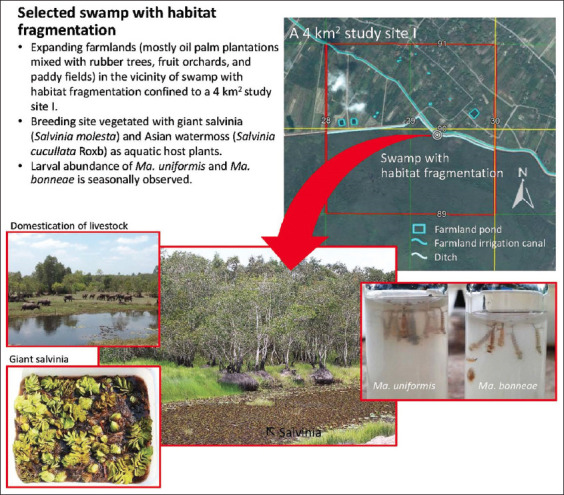
Study site I and the location of selected swamp with habitat fragmentation and aquatic vegetation for *Mansonia* mosquito sampling.

Based on the environmental surveys conducted between 2014 and 2015, a 4 km^2^ survey farmland area was selected as study site I (Figure-1) situated in Tak Bai District, Narathiwat Province, Southern Thailand. *B. malayi* is known to be endemic. It covered a low densely populated land area (<50 persons/1 km^2^) of low-lying farmlands in the vicinity of the Phru To Daeng swamp with habitat fragmentation [24] as a result of the expansion of oil palm plantations mixed with paddy fields, rubber plantations, fruit orchards, built-up water storage systems (farmland irrigation canals, ditches, and farmland ponds), and domestication of animals (livestock and pet animals rather than poultry). The selected swamp with habitat fragmentation, permanently vegetated with giant salvinia (*S. molesta*) and Asian watermoss (*S. cucullata* Roxb), was located at UTM coordinates, 47 N 829319 m E and 689928 m N at an altitude of 10 m (Figure-1).

**Figure-2:**
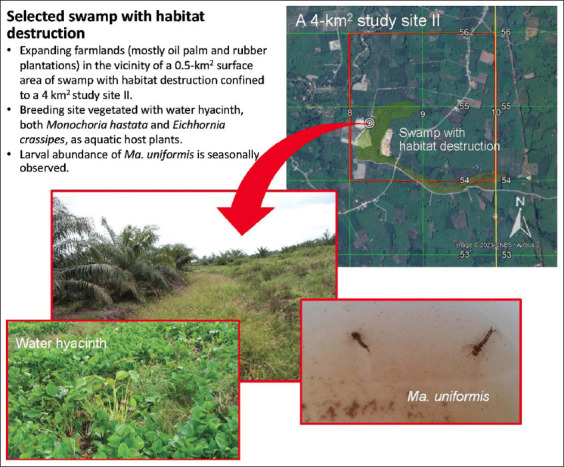
Study site II and the location of selected swamp with habitat destruction and aquatic vegetation for *Mansonia* mosquito sampling.

Based on the environmental surveys conducted between 2014 and 2015, a 4 km^2^ survey farmland area was selected as the study site II (Figure-2) was situated in Thachana District, Suratthani Province, Southern Thailand. It is endemic to *B. malayi*. It covered a low densely populated land area (<50 persons/1 km^2^) of elevated farmlands in the vicinity of “Phru Nam Dam” swamp with habitat destruction [39] as the result of the extensive expansion of rubber plantations mixed with oil palm plantations, fruit orchards, and domestication of pet animals (dogs rather than cats). The selected swamp with habitat destruction that was permanently vegetated with water hyacinth was located at UTM coordinates, 47 P 508276 m E and 1054777 m N at an altitude of 40 m (Figure-2).

**Figure-3:**
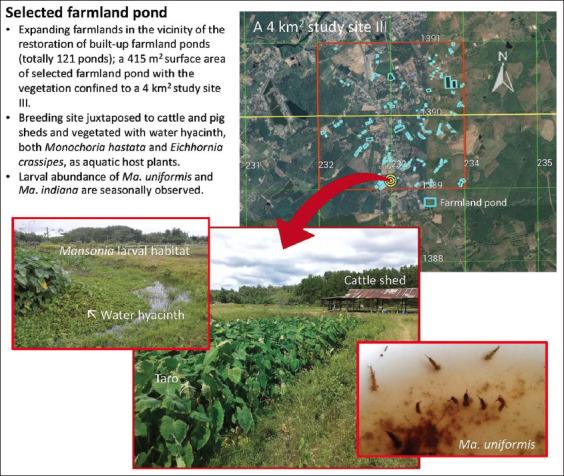
Study site III and the location of selected farmland pond with the restoration and aquatic vegetation for *Mansonia* mosquito sampling.

Based on environmental surveys conducted between 2016 and 2017, a 4 km^2^ survey farmland area was selected as study site III (Figure-3) situated in Borai District, Trat Province, Eastern Thailand. It covered a low densely populated land area (<50 persons/1 km^2^) of low-lying farmland in the vicinity of the restoration of farmland ponds (totaling 121 ponds) because of the expansion of rubber plantations mixed with oil palm plantations and horticulture and the domestication of animals (livestock, pet animals, and poultry). The selected farmland pond permanently vegetated with water hyacinth was located at UTM coordinates 48 P 232986 m E and 1389109 m N, at an altitude of 30 m (Figure-3).

**Figure-4:**
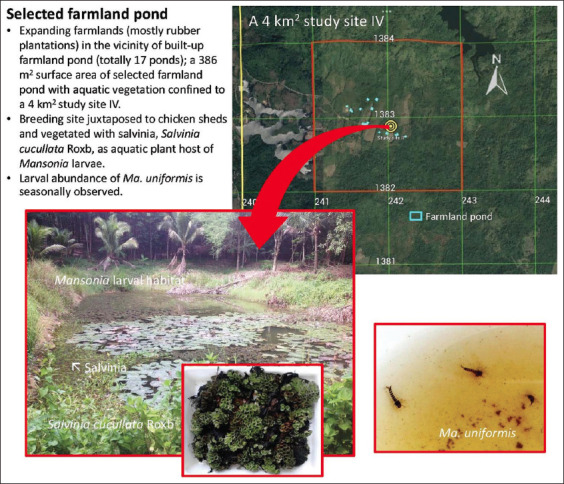
Study site IV and the location of selected farmland pond with the restoration and aquatic vegetation for *Mansonia* mosquito sampling.

Based on the environmental surveys conducted between 2016 and 2017, a 4 km^2^ survey farmland area was selected as study site IV (Figure-4) situated in Borai District, Trat Province, Eastern Thailand. It covered a very low densely populated land area (<10 persons/1 km^2^) of elevated farmland connected to forests and reservoirs in the vicinity of the restoration of farmland ponds (a total of 17 ponds) as a result of the extensive expansion of rubber plantations mixed with fruit orchards and the domestication of animals (poultry and pet dogs, not cats). The selected farmland pond permanently vegetated with Asian watermoss was located at UTM coordinates 48 P 242041 m E and 1382880 m N, at an altitude of 45 m (Figure-4).

### Mosquito collection and identification

Approximately 2–4 sampling sites were located within 10–1000 m of the selected swamps (Figures-1 and 2) or farmland ponds (Figures-3 and 4) at each study site. Sampling sites with well-trained collectors (one collector per sampling site) were used to collect wild-caught *Mansonia* female adult mosquitoes by well-trained collectors. Human landing catch (HLC) collection method was performed using a handheld aspirator throughout the study to collect mosquito population samples between 18:00 h and 21:00 h as the peak of biting activity of *Mansonia* [24, 39] for three consecutive nights. A 45-min landing catch was performed every hour, followed by a 15-min cessation period. Temperature and humidity were also recorded. The HLC collection method was also applied to periodically assess species compositions and abundances between dry and wet seasons at each study site. For this purpose, two wild-caught population samples were collected twice during the dry or wet seasons. Study site I had a dry season (February–May) and a wet season (June–January) [40]. Study sites II–IV had dry season months (November–April) and wet season months (May–October) [41, 42]. Moreover, all wild-caught mosquitoes per hour were placed into plastic cups covered with nylon, transferred to the field-based station, and subsequently kept under suitable conditions with 10% sugar solution. Two entomological experts blindly performed the taxonomic identification of individual female adult mosquitoes as well as larvae [43, 44] under a stereomicroscope.

### Measurement of species composition and abundance

Species richness is represented by the number of different species that can infest each study site and were collected from all sampling sites within each study site at each HLC collection time point. The population ratio (*p_i_*) was expressed as the total number of wild-caught female adult mosquitoes for all *Mansonia* species in wild-caught female adult mosquitoes for each species. The abundance of wild-caught mosquitoes of *Mansonia* spp. or their counterpart species was expressed as man landing rate (MLR), which infers the number of wild-caught female adult mosquitoes of *Mansonia* species per person per hour for each study site.

### Statistical analysis

Population dispersion (distribution of individual species) and population decline (reduction of population size of individual species) of locally adapted *Mansonia* faunas were assessed at a particular point in time in the four geographically defined study sites. To determine population dispersion or decline, the species composition (values of *p_i_*) or abundance (values of MLR) of a given *Mansonia* species were measured according to season variations. In a given *Mansonia* species found in a study site, mean *p_i_* or mean MLR that was derived from 2-time point intervals was compared using a bar graph and error bars (± = 1 SD). The independent samples Mann–Whitney *U*-test (p < 0.05) was used to test whether two population samples of a given *Mansonia* species that were periodically assessed between seasons were likely to derive from the same population (i.e., the two populations have the same distribution of *p_i_* or MLR values).

This study compared the *p*_i_ value of a given *Mansonia* species over change in time to determine changes in the population numbers of *Mansonia* fauna locally adapted to the ever-changing environment of the disturbed swamps within the study sites in Suratthani (study site II) and Narathiwat (study site I) over 5–15 years. To determine population change over a 5-year follow-up, this study employed a 2008 dataset previously published by Yodmek *et al*. [39] by comparing a 2014–2015 dataset for measuring the *p_i_* of a given *Mansonia* dominant vs. counterpart species that was found in the vicinity of the Phru Nam Dam swamp with habitat destruction as study site II as mentioned earlier. A 2000–2001 dataset previously published by Apiwathnasorn *et al*. [24] as a reference was used to compare a 2014–2015 dataset for measuring the *p_i_* of a given *Mansonia* dominant vs. counterpart species that was found in the vicinity of the “Phru Toh Daeng” swamp with habitat fragmentation as study site I to determine the population change over a 15-year follow-up. This did not permit the analysis of the population change of a given *Mansonia* species found in the vicinity of built-up farmland ponds with restoration and aquatic vegetation as the study sites in Trat because there was no reference in this study investigation.

Based on a two-point straight line graph using the *p_i_* values for a given *Mansonia* species observed between the study site and reference during the study, the following types of straight lines were interpreted. If there is a trend of increasing the number of a given *Mansonia* species as the dominant species (≥50% or more of the *Mansonia* faunas), the bold straight line indicates that the *p_i_* of that locally adapted species found in the study site is greater than that found by the reference species. If there is a trend of decreasing or staticizing the number of a given *Mansonia* species as a counterpart species (<50% of *Mansonia* faunas), the dashed straight line indicates that the *p_i_* of that locally adapted species found in the study site is less than that found by the reference species. If there is a trend of the disappearance of a given *Mansonia* species, a thin straight line indicates the value of zero *p_i_* of that species not locally adapted to the local environment of the study site or that observed by the reference.

Simple linear regression was used to estimate the slope (unstandardized coefficient beta) of the two-point data with time (independent variable) as the x-intercept and *p_i_* (dependent variable) as the y-intercept. If these two variables are probabilistically related, then there is uncertainty in the *p_i_* value over time for a fixed period. The slope is a measure of how steep the linear regression line is, that is, indicating the state of sharply or dramatically increasing/decreasing the population of a given *Mansonia* species at a certain point in time. In this study, the negative or positive slope permitted the determination of the sudden or gradual changes in *p_i_* for a given *Mansonia* species over time.

## Results

### Assessment of species composition

**Figure-5:**
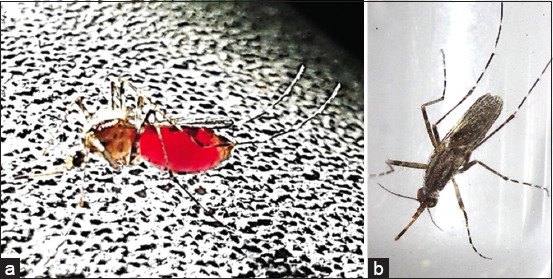
*Mansonia uniformis* as representative of dominant species locally adapted to all study sites. (a) In site III, immediately after taking cow blood meal in cattle shed during the night time (Figure-3), the fully engorged female adult mosquito of *Ma. uniformis* digested blood while resting on bamboo culm. (b) Morphologic characteristics of *Ma. uniformis* female that was wild caught by human landing catch was taxonomically identified under stereomicroscope and was compared well to that fully engorged female in A.

Of the 1087 wild-caught *Mansonia* mosquitoes collected by HLC from study sites I (387), II (237), III (487), and IV (56), six species of *Mansonia* were found in this study. Among the six species of *Mansonia* being identified, *Ma. uniformis* was the dominant species (Figure-5) that constituted the highest proportion (≥50%) of *Mansonia* faunas commonly found in all the study sites: I (n = 182, *p_i_* = 0.470), II (n = 167, *p_i_* = 0.705), III (n = 372, *p_i_* = 0.764), and IV (n = 37, *p_i_* = 0.661) (Supplementary Table-1).

**Supplementary Table-1:** Species and number^a^ of *Mansonia* wild-caught by HLC in four different study sites.

Species	Site I	Site II	Site III	Site IV
All	387	237	487	56
*Ma. uniformis*	182 (0.470)	167 (0.705)	372 (0.764)	37 (0.661)
*Ma. indiana*	15 (0.039)	36 (0.152)	97 (0.199)	1 (0.018)
*Ma. annulifera*	0	0	4 (0.008)	6 (0.107)
*Ma. annulata*	58 (0.150)	0	14 (0.029)	4 (0.071)
*Ma. bonneae*	116 (0.300)	34 (0.143)	0	3 (0.054)
*Ma. dives*	16 (0.041)	0	0	5 (0.089)

a Including those observed in dry and wet seasons. Population ratio (*p_i_*) in parentheses.

Study site I elicited five species of *Mansonia* fauna (n = 387) with variable proportions: *Ma. uniformis* (n = 182, *p_i_* = 0.470), *Ma. bonneae* (n = 116, *p_i_* = 0.300), *Ma. annulata* (n = 58, *p_i_* = 0.150), *Ma. dive*s (n = 16, *p_i_* = 0.041), and *Ma. indiana* (n = 15, *p_i_* = 0.039)*. Ma. annulifera* was the only counterpart species that disappeared during the seasons observed by the study.

Only three species of *Mansonia* fauna (n = 237) with variable proportions were established at study site II: *Ma. uniformis* (n = 167, *p_i_* = 0.705), *Ma. indiana* (n = 36, *p_i_* = 0.152), and *Ma. bonneae* (n = 34, *p_i_*= 0.143. The remainder *(Ma. annulifera, Ma. annulata*, and *Ma. dives*) disappeared during the seasons observed in this study.

Study site III elicited four species of *Mansonia* fauna (n = 487) with variable proportions: *Ma. uniformis* (n = 372, *p_i_* = 0.764), *Ma. indiana* (n = 97, *p_i_*= 0.199), *Ma. annulata* (n = 14, *p_i_* = 0.029), and *Ma. annulifera* (n = 4, *p_i_* = 0.008). *Ma. bonneae* an*d Ma. dives* disappeared during the seasons observed in this study.

Study site IV established all six species of *Mansonia* fauna (n = 56) with variable proportions: *Ma. uniformis* (n = 37, *p_i_* = 0.661), *Ma. annulifera* (n = 6, *p_i_* = 0.107), *Ma. dive*s (n = 5, *p_i_* = 0.089), *Ma. annulata* (n = 4, *p_i_* = 0.071), *Ma. bonneae* (n = 3, *p_i_* = 0.054), and *Ma. indiana* (n = 1, *p_i_* = 0.018).

**Figure-6:**
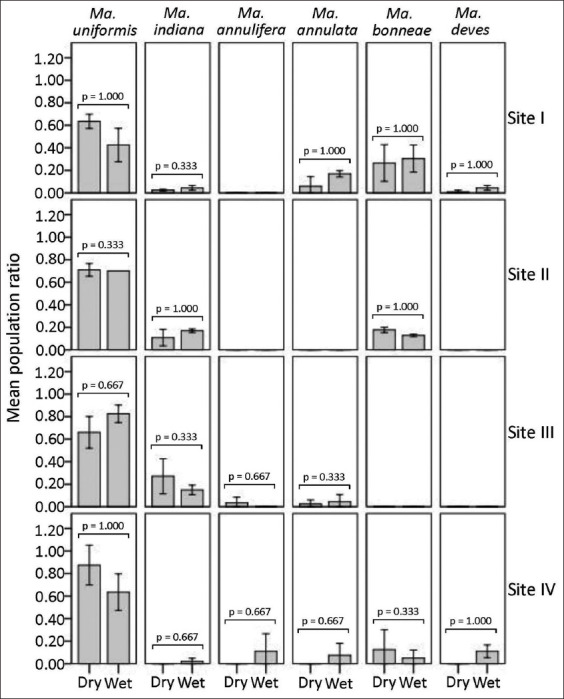
Mean population ratios of *Mansonia* faunas that were periodically assessed by season among four different study sites. Error bars (±1 SD) are shown. There was no significant difference in the distribution of the population ratio (*p_i_*) values of each *Mansonia* species measured between seasons (two independent samples Mann–Whitney *U*-test, p > 0.05) in any study site.

Figure-6 shows the aggregate distribution of *Mansonia* species locally adapted to the environments of four distinct study sites based on the periodic assessment of species compositions by season. There were no significant differences (two independent samples Mann–Whitney *U*-test, p < 0.05) in the distribution of the mean *p_i_* values for any *Mansonia* species measured between dry season and wet season months with respect to the population dispersion of locally adapted *Mansonia* faunas found in four different study sites.

### Assessment of seasonal abundance

**Figure-7:**
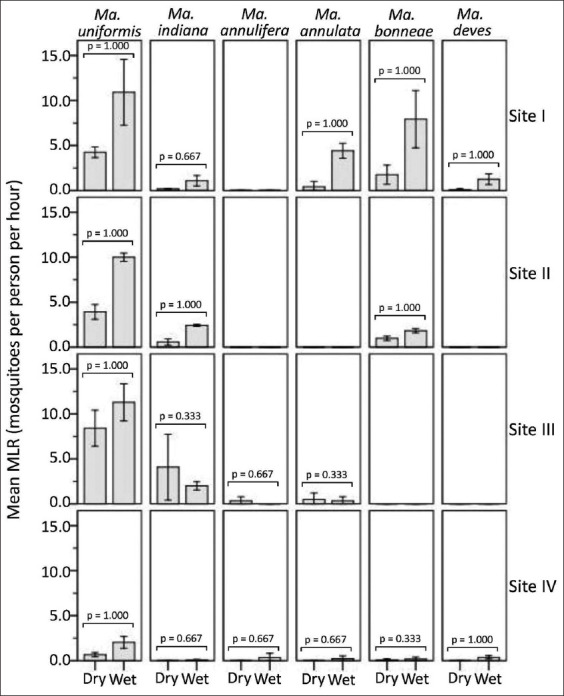
Mean man landing rate (MLR) for *Mansonia* faunas that were periodically assessed by season among four different study sites. Error bars (±1 SD) are shown. There was no significant difference in the distribution of the MLR values for each *Mansonia* species measured between seasons (two independent samples Mann–Whitney *U*-test, p > 0.05) in any study site.

Based on periodic assessment of seasonal abundances measured in terms of mean MLR, *Mansonia* faunas locally adapted to the environments of four distinct study sites I to IV were distinguishable from each other but likely season-dependent (Figure-7).

The mean MLRs (wet vs. dry) for *Ma. uniformis* (10.92 vs. 4.25), *Ma. bonneae* (7.92 vs. 1.75), *Ma. annulata* (4.42 vs. 0.42), *Ma. dives* (1.25 vs. 0.08), and *Ma. indiana* (1.08 vs. 0.17) at study site I were observed. In contrast, the mean MLRs of *Ma. uniformis* (10.0 vs. 3.92), *Ma. indiana* (2.42 vs. 0.58), and *Ma. bonneae* (1.84 vs. 1.0) were higher at study site II. Site III elicited higher mean MLRs for *Ma. uniformis* in the wet season than in the dry season (11.29 vs. 8.42). On the other hand, more abundant counterpart species, *Ma. indiana* (2.0 vs. 4.0), *Ma. annulata* (0.5 vs. 0.34), and *Ma. annulifera* (0.34 vs. zero), were observed in the dry season compared with the wet season. Contrary to study site III, study site IV exhibited higher mean MLRs during the wet season than during the dry season, especially for *Ma. uniformis* (2.03 vs. 0.67) and *Ma. bonneae* (0.16 vs. 0.08). Remarkably, the following four counterpart species with variable mean MLRs were abundant during the wet season: *Ma. annulifera* (0.34), *Ma. dives* (0.36), *Ma. annulata* (0.22), and *Ma. indiana* (0.06).

With respect to the seasonal abundances of locally adapted *Mansonia* faunas found in all the study sites (Figure-7), there were no significant differences (two independent samples Mann–Whitney *U* test, p > 0.05) in the distribution of MLR values for any *Mansonia* species measured between the wet season and dry season months.

### Measurement of *Mansonia* population change

In the simple linear regression and slope analysis, the population ratios (*p_i_* values) of locally adapted *Mansonia* dominant vs. counterpart species that occupy the changing habitat were assessed over the change in time based on the data measured by 2-time point intervals for Narathiwat (observed by the reference vs. study site I) and Suratthani (observed by the reference vs. study site II).

**Figure-8:**
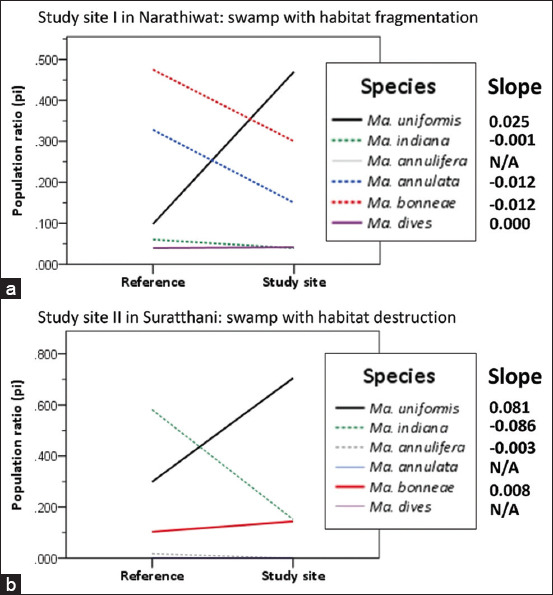
The slope (unstandardized coefficient beta) calculated for a simple linear regression line of two variables related. In this study, the negative or positive slope permitted the determination of the population change, that is, the population ratios for a given *Mansonia* species (dependent variable) locally adapted to the swamp with habitat fragmentation in Narathiwat (a) and the swamp with habitat destruction in Suratthani (b) were dependent on time (independent variable). Time points for measuring the *p_i_* value for a given *Mansonia* species were used for Suratthani (between a 5-year follow-up by the study site I and a year-zero study by the reference) and Narathiwat (between a 15-year follow-up by the study site II and a year-zero study by the reference) as described in the text. N/A - data not applicable for analysis.

A remarkable population decline was observed in Narathiwat, where changes in the populations of *Mansonia* faunas locally adapted to the swamp with habitat fragmentation were assumed (Figure-8a and Supplementary Table-2 [24, 39]), except for *Ma. uniformis*. *Ma. uniformis* is an emerging dominant species found in study site I. The species exhibited a large proportion (*p_i_* = 0.470) of *Mansonia* faunas, showing a dramatic increase (unstandardized coefficient beta = 0.025) over a 15-year follow-up period. On the other hand, both *Ma. annulata* and *Ma. bonneae* tended to dramatically decrease population ratios with the same slope (unstandardized coefficient beta = −0.012).

**Supplementary Table-2:** The population dispersion and decline of locally adapted *Mansonia* faunas in the vicinity of the disturbed swamps in Narathiwat and Suratthani observed by the reference and this study.

Species	No. (*p*_i_) of wild-caught *Mansonia* mosquitoes					

	Reference I^a^ (N=1,129)	Site I (N=387)	Slope	Reference II^b^ (N=1,030)	Site II (N=237)	Slope
*Ma. uniformis*	117 (0.098)	182 (0.470)	0.025	308 (0.299)	167 (0.705)	0.081
*Ma. indiana*	72 (0.060)	15 (0.039)	-0.001	598 (0.581)	36 (0.152)	-0.086
*Ma. annulifera*	0	0	N/A	18 (0.017)	0	-0.003
*Ma. annulata*	391 (0.328)	58 (0.150)	-0.012	0	0	N/A
*Ma. bonneae*	566 (0.475)	116 (0.300)	-0.012	106 (0.103)	34 (0.143)	0.008
*Ma. dives*	46 (0.039)	16 (0.041)	0.000	0	0	N/A

Using a 2000-2001 dataset published by reference I [24]

a , and a 2008 dataset published by reference II [39]

b . Unstandardized coefficient beta (B) for simple linear regression. N/A – not applicable.

Similarly, in Suratthani, a remarkable decline in the populations of *Mansonia* faunas locally adapted to the swamp with habitat destruction is seen in Figure-8b and Supplementary Table-2, except for *Ma. uniformis*. *Ma. uniformis* is also an emerging dominant species found in study site I, exhibiting a great proportion (*p_i_* = 0.705) of *Mansonia* faunas. A sharp increase (unstandardized coefficient beta = 0.081) was observed in this species over a 5-year follow-up period. However, there was a dramatic decrease in the population ratios of *Ma. indiana* (unstandardized coefficient beta = −0.086) and *Ma. annulifera* (unstandardized coefficient beta = −0.003).

## Discussion

As a result of the expansion of farmlands by farmers and landowners, the first question arises as to how the expansion of farmlands that invaded swamps as freshwater habitats of *Mansonia* vectors had the expected effects on population dispersion and the decline of locally adapted *Mansonia* faunas. Expansion of farmlands with animal domestication in the vicinity of low-lying versus elevated disturbed swamps had negative effects on reduced species richness of *Mansonia* faunas, leading to population dispersion of locally adapted *Mansonia* faunas with aggregate distribution. This may be explained by the aggregation of locally adapted *Mansonia* faunas when foraging for blood meal sources and co-breeding in their habitats, as seen in this study and elsewhere in Asia [24, 45], Africa [46], and South America [47].

Study site I covered expanding farmlands with livestock domestication and pet animals in the vicinity of low-lying swamps with habitat fragmentation and aquatic vegetation. Although water storage systems were available, *Mansonia* preferred breeding in disturbed swamps. The observations exhibited more species richness (five species) of locally adapted *Mansonia* faunas *(Ma. uniformis*, *Ma. bonneae*, *Ma. annulata*, *Ma. dives*, and *Ma. indiana*) with variable proportions and seasonal abundances (Figures-6 and-7). As shown in Figure-6, although season variation was ecologically constrained, the population ratios of locally adapted *Mansonia* faunas were probably staticized between seasons by maintaining the numbers of dominant vs. counterpart species. The abundance of locally adapted *Mansonia* faunas was probably season-dependent (Figure-7). In Figure-8a, it was noticed that, except for *Ma. uniformis*, the population decline of locally adapted *Mansonia* faunas over a 15-year follow-up was affected by the negative effects of the expanding farmlands with domestication of animals (livestock and pet animals) in the vicinity of the swamp with habitat fragmentation and aquatic vegetation. *Ma. uniformis* exhibited a great proportion (about 50%) of locally adapted *Mansonia* faunas, or 5-fold greater than its proportion (about 10%) observed by the reference [24], but likely shared the biological and physiological conditions with its four counterpart species (*Ma. bonneae*, *Ma. annulata*, *Ma. dives*, and *Ma. indiana*) that can infest in the swamp with habitat fragmentation.

Study site II covered expanding farmlands with the domestication of pet animals in the vicinity of an elevated swamp with habitat destruction and aquatic vegetation. *Mansonia* preferred to breed in disturbed swamps, even though there was a water storage system. The observations showed lower species richness (three species) of locally adapted *Mansonia* faunas*(Ma. uniformis*, *Ma. indiana*, and *Ma. bonneae*) but similar patterns of variable proportions and seasonal abundances (Figures-6 and-7). In Figure-8b, it was also noticed that, excepting *Ma. uniformis*, the population decline of locally adapted *Mansonia* faunas over a 5-year follow-up was affected by the negative effects of the expanding farmlands with domestication of animals (pet animals) in the vicinity of the swamp with habitat destruction and aquatic vegetation. *Ma. uniformis* exhibited a great proportion (about 70%) of locally adapted *Mansonia* faunas, or 2.5-fold greater than its proportion (about 30%) observed by the reference [39], but likely shared the biological and physiological conditions with its counterpart species (*Ma. indiana* and *Ma. bonneae*) that can breed in the swamp with destruction. *Ma. annulifera*, *Ma. dives*, and *Ma. annulata* were among the counterpart species that were likely to be sensitive to such ecological vulnerability of the swamp with habitat destruction.

These findings observed at study sites I and II compared well with previous findings in Indonesia [5, 9, 10, 16, 17, 45]. Pratiwi *et al*. [10] demonstrated that a 2017–2018 dataset of *Mansonia* vector populations observed in two distinct *B. malayi* endemic villages in Banyuasin Regency, South Sumatra, Indonesia, had a variety of locally adapted *Mansonia* faunas, showing variable proportions and seasonal abundances of *Ma. uniformis*, *Ma. annulifera*, *Ma. indiana*, *Ma. annulata*, *Ma. bonneae*, and *Ma. dives*. *Ma. uniformis*, *Ma. annulifera*, and *Ma. indiana* constituted about 50% of *Mansoni*a faunas in endemic villages in the vicinity of swamps. However, *Ma. bonnea*e, *Ma. uniformis*, and *Ma. dives* constituted approximately 51% of *Mansonia* faunas in *B. malayi* endemic villages in the vicinity of *Mansoni*a-infested plantations and paddy fields. Ridha *et al*. [9] showed that in a 2017 dataset of *Mansonia* vector populations observed in *B. malayi* endemic villages in the vicinity of swamps in Hulu Sungai Utara, South Kalimantan, Indonesia, less species richness constituted only *Ma. dives* (*p_i_* = 0.598) and *Ma. uniformis* (*p_i_* = 0.402). Mulyaningsih *et al*. [45] reported that in a 2018 dataset of *Mansonia* vector populations observed in *B. malayi* endemic villages in the vicinity of swamps in Banyuasin, South Sumatera, Indonesia, locally adapted *Mansonia* faunas constituted mostly *Ma. uniformis* (*p_i_* = 0.617), *Ma. annulifera* (*p_i_* = 0.368), and *Ma. dives* (*p_i_* = 0.015). Conversely, in the *B. malayi* endemic villages of Musi Rawas, South Sumatera, Indonesia, only *Ma. uniformis* was observed [5]. However, these studies did not evaluate the functions of swamps with habitat fragmentation/destruction or the anticipated effects of expanding farmlands in the vicinity of disturbed swamps infested with *Mansonia* mosquitoes. In contemporarily *B. malayi* endemic countries in Southeast Asia, *Ma. uniformis* is well adapted to swamp ecosystems that have changed over time, although ever-expanding farmlands or human settlements have invaded its habitat. These anticipated effects might have affected the population decline in locally adapted *Mansonia* faunas and, eventually, could alleviate the interruption of local *B. malayi* transmission in endemic areas targeted by the lymphatic filariasis elimination program in the absence of vector control measures [48–50].

This study explored the anticipated effects of expanding farmlands that established farmland ponds to provide a variety of freshwater habitats for the *Mansonia* fauna. Similar to that observed at study sites I and II, two study sites III and IV demonstrated that the effects of expanding farmlands with the domestication of animals in the vicinity of low-lying ponds versus elevated farmland ponds with the restoration and aquatic vegetation affected the aggregated population dispersion of locally adapted *Mansonia* fauna.

The study site III covered expanding farmlands with livestock and pet animals domesticated in the vicinity of low-lying farmland ponds with restoration and aquatic vegetation. We observed lower species richness (four species) of locally adapted *Mansonia* faunas *(Ma. uniformis*, *Ma. indiana*, *Ma. annulata*, and *Ma. annulifera*) with variable proportions (Figure-6) but likely higher seasonal abundances (Figure-7). Study site IV covered expanding farmland with the domestication of pet animals in the vicinity of elevated farmland ponds with restoration and aquatic vegetation. We observed higher species richness (six species) of locally adapted *Mansonia* faunas *(Ma. uniformis*, *Ma. annulifera*, *Ma. bonneae*, *Ma. dives*, *Ma. annulata*, and *Ma. indiana*) with variable proportions (Figure-6) but likely lower seasonal abundances (Figure-7). In both study sites, *Ma. uniformis* exhibited a proportion of as high as 75% of locally adapted *Mansonia* faunas that were remarkably regulated by the wet season. *Ma. uniformis*, *Ma. indiana*, *Ma. annulata*, and *Ma. annulifera* are indicator species of the establishment or re-establishment of locally adapted *Mansonia* faunas if accompanied by the ecological vulnerability of the farmland pond ecosystem. *Ma. bonneae* and *Ma. dives* were more likely to be indicator species that were locally adapted to expanding farmlands connected to the forest, as observed in study site IV.

## Conclusion

This study provided proof that expanding farmlands with domestication of animals (livestock rather than pet animals) either in the vicinity of disturbed swamps with aquatic vegetation or in the vicinity of built-up farmland ponds with restoration and aquatic vegetation had anticipated effects on the aggregated distribution of the populations of locally adapted *Mansonia* faunas, that is, species compositions and abundances shaped by local environments of four different geographically defined study sites. Similar to other faunas that negatively affect expanding farmlands in rural and urban settings [51, 52], locally adapted *Mansonia* faunas represent a diverse assemblage of *Mansonia* species with different adaptations, ecological tolerances, and host exploitation strategies in life. These changes in *Mansonia* ecology of expanding farmland, whether they invaded or established habitats, depend on the function of disturbed freshwater swamps or built-up farmland ponds, whether they provide a variety of freshwater habitats for breeding. Unless the disturbed swamps are permanently saturated with water from surface runoff and are vegetated with aquatic host plants, the population decline of locally adapted *Mansonia* faunas will be affected by the loss of function favoring *Mansonia* breed. In addition, built-up farmland ponds can favor the population dispersion of locally adapted *Mansonia* faunas if they permanently collect and store surface runoff and are vegetated with aquatic host plants. In the case of the domestication of animals, the expansion of farmland in the vicinity of either disturbed swamps or built-up farmland ponds provided a variety of sources of blood feed for animals, thus maintaining population dispersion. Taken together, drought mitigation stakeholders, including farmers, landowners, and other allied sectors, can build resilience for livestock, crops, and people’s livelihoods. However, attention should be paid to the advantages and disadvantages of the restoration and aquatic vegetation of such farmland ponds, as well as the domestication of livestock and/or pet animals in farmland areas where *Mansonia* can infest or reinfest.

## Data availability

The supplementary data can be available from the corresponding author upon a reasonable request.

## Authors’ Contributions

AB and SP: Conceived the study concept and design, methodology and data collection, data presentation, data analysis, original artworks or graphic illustration, and edited the manuscript. AB, SP, AI, and WR: Leveraged the data of *Mansonia* faunas obtained from four different study sites and drafted the manuscript. All authors have read, reviewed, and approved the final manuscript.
